# Downregulation of PRMT1 promotes the senescence and migration of a non-*MYCN* amplified neuroblastoma SK-N-SH cells

**DOI:** 10.1038/s41598-018-38394-6

**Published:** 2019-02-11

**Authors:** Yu-Jen Lee, Wen-Wei Chang, Chien-Ping Chang, Tsung-Yun Liu, Chun-Yi Chuang, Kun Qian, Y. George Zheng, Chuan Li

**Affiliations:** 1 Department of Biomedical Sciences, Chung Shan Medical University, Taichung, Taiwan Republic of China; 2 Department of Medical Research, Jen-Ai Hospital, Taichung, Taiwan Republic of China; 3 Department of Medical Research, Chung Shan Medical University Hospital, Taichung, Taiwan Republic of China; 4 School of Medicine, Chung Shan Medical University, Taichung, Taiwan Republic of China; 5 Department of Otolaryngology, Chung Shan Medical University Hospital, Taichung, Taiwan Republic of China; 6 Department of Pharmaceutical & Biomedical Sciences College of Pharmacy, University of Georgia, Athens, Georgia USA

## Abstract

Protein arginine methyltransferase 1 (PRMT1) catalyzing the formation of asymmetric dimethylarginines has been implicated in cancer development, metastasis, and prognosis. In this study, we investigated the effects of low PRMT1 levels on a non-*MYCN* amplified neuroblastoma SK-N-SH cell line. Stable *PRMT1*-knockdown (*PRMT1*-KD) cells showed reduced growth rates and cell cycle arrest at G_2_/M. They also exhibited senescent phenotypes and increased p53 expression. p21 and PAI-1, which are two p53 downstream targets critical for senescence, were significantly induced in SK-N-SH cells subjected to either *PRMT1*-KD or inhibitor treatment. The induction was suppressed by a p53 inhibitor and marginal in a p53-null SK-N-AS cell line, suggesting dependence on p53. In general, the DNA damage and ROS levels of the *PRMT1*-KD SK-N-SH cells were slightly increased. Their migration activity also increased with the induction of PAI-1. Thus, PRMT1 downregulation released the repression of cellular senescence and migration activity in SK-N-SH cells. These results might partially explain the poor prognostic outcome of low PRMT1 in a non-*MYCN-*amplified cohort and indicate the multifaceted complexity of PRMT1 as a biological regulator of neuroblastoma.

## Introduction

Protein arginine methylation is a posttranslational modification implicated in signal transduction, transcriptional regulation, DNA repair and RNA processing^1,2^. Protein arginine methyltransferase 1 (PRMT1) is the first identified and the most predominant PRMT^3,4^. PRMT1, along with PRMT 2, 3, 4, 6 and 8, belong to the type I enzymes that catalyze the formation of asymmetric ω-*N*^*G*^, *N*^*G*^ dimethylarginine (ADMA). PRMT5 and 9 are type II enzymes that catalyze the formation of symmetric ω-*N*^*G*^, *N*^*G’*^ dimethylarginine. PRMT7 is the only type III PRMT that catalyzes the formation of ω- *N*^*G*^ monomethylarginine^1,2,5^.

PRMT1 plays roles in various cellular processes. Numerous nucleic acid binding proteins serve as PRMT1 substrates and the methylation may affect subcellular localization or RNA binding activities of the modified proteins^6,7^. The early identification of PRMT1 as an interacting partner of the cytoplasmic domain of IFNα receptor^8^ and the subsequent demonstration of the involvement of PRMT1 in STAT1/PAIS1^9,10^, lymphocyte signaling^11^, and TNFα/NF-κB signaling^12^ suggest that PRMT1 participates in immune response signaling. Besides, PRMT1 is involved in Akt signaling because its methylation of forkhead transcription factor FOXO1 counteracts Akt phosphorylation^13^. PRMT1 can function as a coactivator of the epigenetic regulation of the histone code via the asymmetric dimethylation of histone H4 Arg-3 (H4R3me2a)^14,15^. The methylation of MRE11 and 53BP1 by PRMT1 indicates that this enzyme is implicated in DNA damage response^16–18^.

The failure of homozygous mouse *prmt1* mutant embryos to develop shortly after implantation supports a fundamental role for PRMT1^19^. The loss of PRMT1 in mouse embryonic fibroblasts (MEFs) results in spontaneous DNA damage, cell cycle progression delay, checkpoint defects, aneuploidy, and polyploidy, indicating that PRMT1 is essential for genome integrity and cell proliferation^20^. We knocked down *prmt1* via antisense morpholino (AMO) injections in zebrafish embryos and showed defective convergence and extension during gastrulation. This knockdown also affects embryonic brain development^21^. Mutant mice with *prmt1* specifically knocked out in the central nervous system (CNS) show post-natal growth retardation with tremors, with mice dying two weeks after birth. This mouse model suggests specific roles of PRMT1 in the nervous system^22^.

We studied the genetic variations and mutations in Hirschsprung disease (HSCR) or aganglionic megacolon, a congenital disorder frequently encountered in pediatric surgery^23,24^. Using tissue samples from patients with HSCR, we showed the distribution of human PRMT1 in neurons in the submucosal and myenteric plexuses of the enteric nervous system, which is the largest group in the peripheral nervous system (PNS)^25^. In patients with HSCR, the absence of enteric neurons derived from migratory neural crest cells in the distal intestine results in coordination problems of smooth muscle contractions and finally causes intestinal obstruction. Neural crest cells must undergo epithelial mesenchymal transition (EMT), which is similar to EMT in cancer metastasis, to interact with a microenvironment and reach their final destination^26^. Neuroblastoma is an extracranial solid pediatric tumor arising from the developing neural crest along its migratory pathways and accounts for 7% of the total tumors observed in children^27^. The increased expression and involvement of PRMT1 have been reported in various cancers including bladder^28^, liver^29^ esophageal^30^ and head and neck cancer^31^. As such, we aimed to study PRMT1 in neuroblastoma, a tumor derived from the neural crest cells.

Early experiments showed that PRMT1 is required for the neuronal differentiation potential of the cancer cells derived from neural crest cells. Suppressing PMRT1 inhibits neurite outgrowth in rat adrenal medulla pheochromocytoma PC12 cells, which are also derived from neural crest cells^32^. Knockdown of PRMT1 in mouse Neuro2a neuroblastoma cells also greatly reduces the percentage of neurite-bearing cells^33^. For human neuroblastoma, the amplification of the *MYCN*(V-myc myelocytomatosis viral-related oncogene, neuroblastoma derived [avian]) oncogene occurs in 20–25% of the patients, and the degree of amplification is associated with advanced stage and poor prognosis^27^. PRMT1 is a direct target of *MYCN* in *MYCN*-amplified or overexpressed neuroblastoma^34^. On the other hand, PRMT1 can modify MYCN and affect its stability. PRMT1 siRNA knockdown reduces MYCN expression and neuroblastoma cell viability^35^. However, over 50% of high-risk patients are without MYCN overexpression or amplification. Neuroblastoma cell lines can be divided into N-, S-or I-type based on their morphological and biochemical characters. N-type neuroblastic cells have long neuritic processes, S-type cells are adherent to the substratum cells with an epithelial-like morphology and I-type cells are intermediate^36^. In our study, we used the S-type neuroblastoma cell line SK-N-SH to investigate the roles of PRMT1 in neuroblastoma.

## Materials and Methods

### Cell culture and treatments

Human SK-N-SH and SK-N-AS neuroblastoma cells were grown in DMEM medium (Gibco/Life Technologies) supplemented with 10% fetal bovine serum (FBS; Hyclone), 100 U/mL of penicillin, 100 µg/mL streptomycin, 2 mM of L-glutamine (Thermo), and 1% non-essential amino acids (Gibco/Life Technologies). Human SK-N-MC neuroblastoma cells were grown in MEM medium (Gibco/Life Technologies) supplemented with 10% FBS, 100 U/mL of penicillin and 100 μg/mL streptomycin. The p53 inhibitor pifithrin-α and the PAI-1 inhibitor PAI-039 were from Sigma.

### Stable shRNA-mediated PRMT1 knockdown in SK-N-SH cells

Lentiviral particles with short hairpin RNA (shRNA) targeting human PRMT1 (A1 with the target sequences: 5′*-*GTGTTCCAGTATCTCTGATTA*-*3′; B1 with the target sequences: 5′*-*CCGGCAGTACAAAGACTACAA*-*3′), and a negative control construct (pLKO_TRC005) were obtained from the National RNAi Core Facility (Academia Sinica, Taiwan). Cells were infected by lentivirus in complete growth medium supplemented with polybrene (Sigma-Aldrich). After 24 h, cells were grown and selected in medium containing 5 µg/ml puromycin (Sigma-Aldrich).

### Cell growth assay and viability assay

For cell growth assay, aliquots of cultured cells were mixed with trypan blue and the cell numbers were counted every 24 h. The alamarBlue cell proliferation assay was used to evaluate cell viabilities. Cells seeded in 96-well plates (10,000 cells/well) were incubated with the alamarBlue solution (Bio-Rad) for 24 h. Absorbance at the wavelength of 570 nm and 600 nm was measured by spectrophotometry after the required incubation time. The viability was calculated as the percentage difference between treated and control cells according to the instructions of the manufacturer.

### Immunofluorescent analysis

The cells were cultured on glass coverslips and fixed with 2% paraformaldehyde (Sigma-Aldrich) in phosphate buffered saline (PBS) at room temperature for 15 min. After washing three times for 3 min with PBS, the fixed cells were permeabilized for 5 min at room temperature with PBS containing 0.5% Triton X-100 and washed again as described above. Blocking was performed with PBS containing 0.01% TritonX-100 and 1% bovine serum albumin (1% BSA/PBS-T) for 60 min at room temperature. The cells were incubated with PBS-T containing primary antibodies (1:200 for phospho-Histone H2AX from Cell Signaling) for at 4 °C for overnight, washed four times for 5 min with PBS-T, and incubated with PBS-T containing FITC-conjugated anti-rabbit antibody (Jackson ImmunoResearch Laboratories, PA, USA) for 1 h, followed by 4′,6-diamidino-2-phenylindole (DAPI; 0.5 μg/ml; Roche) for 10 min in the dark at room temperature. The cells were washed for four times with PBS-T and observed with a fluorescence microscope (ZEISS AXioskop2).

### Immunoblotting

Total cell extracts were separated by SDS-PAGE and subsequently transferred to nitrocellulose membranes (Sartorius Stedim Biotech). The membranes were blocked in 7% skimmed dry milk in TTBS (10 mM Tris-HCl, pH 7.5; 100 mM NaCl; 0.1% Tween 20) for 1 h, incubated with primary antibodies (1:2500 for anti-PRMT1, 1:1000 for anti-ASYM24 from Merck/Millipore; 1: 1000 for anti-ADMA, anti-p53 and anti-p21 from Cell Signaling; 1:1000 for anti-phospho-p53, anti-p38, anti-phospho-p38 and anti-PAI-1 from Santa Cruz; 1:1000 for anti-MYCN from proteintech; 1:10000 for β-actin from Novus) at 4 °C overnight, washed four times for 5 min in TTBS, incubated with secondary antibodies (anti-mouse, rabbit or goat IgG horseradish peroxidase conjugate) for 1 h, and then washed again as described above. Chemiluminescent detection was performed using the VisGlow substrate for HRP (Visual Protein, Taiwan) according to the manufacturer’s instructions.

### Senescence-associated β-galactosidase activity assay

Senescence-associated β-galactosidase (SA-β-gal) activity was measured with a β-galactosidase staining kit (BioVision, Palo Alto, CA, USA) according to the manufacturer’s instructions. Briefly, normal and PRMT1-knockdown cells were cultured on glass coverslips and washed once in PBS, fixed for 15 min at room temperature with 1 mL of fixative solution, washed and incubated overnight at 37 °C with the staining solution mix. Accumulation of a distinctive blue color in senescent cells was then observed by microscopy (total magnification × 400).

### Flow cytometry analysis for cell cycle and ROS detection

For cell cycle analysis, cells were harvested and collected by centrifugation, and then washed with PBS. The cells were resuspended with DNA Staining Solution containing propidium iodide (Sigma-Aldrich) and permeabilization solution in the dark. Intracellular ROS levels of the cells were measured by the fluorescent probe 2′,7′-Dichlorofluorescin diacetate (DCFH-DA, Sigma-Aldrich, USA). After the indicated treatment, cells were trypsinized, collected by centrifugation, and then incubated with serum-free medium containing 10 µM of DCFH-DA at 37 °C for 30 min. After washing three times to remove the uncombined probe, the stained cells were analyzed with Epics XL flow cytometer (Beckman Coulter Inc., Brea, CA, USA). Data were further analyzed with the WinMDI software (National Institutes of Health, Bethesda, MD, USA).

### Quantitative RT-PCR analyses

Total RNA was extracted by NucleoSpin^®^ RNA kit (MACHEREY-NAGEL), and reverse transcribed to cDNA using random primers. SYBR Green-based qPCR reactions for simultaneous detection and quantification of the cDNA samples were performed on an ABI StepOnePlus™ Real-Time PCR System. The cycling conditions were as follows: 50 °C for 2 min, 95 °C for 10 min, followed by 40 cycles of 95 °C for 10 sec and 60 °C for 1 min. The end-point used in the real-time quantification was calculated by the StepOne software (Applied Biosystems), and the threshold cycle number (Ct value) for each analyzed sample was calculated. Each target gene was normalized to GAPDH. The relative gene expression differences between groups were calculated by 2^−ΔΔCt^. The primers used were GAPDH-F:5′- ACCCACTCCTCCACCTTTGA-3′, R: 5′-CTGTTGCTGTAGCCAAATTCG-3′; PRMT1-F: 5′-TGCGGTGAAGATCGTCAAAGCC-3′, R:5′-GACTCGTAGAAGAGGCAGTAG-3′; p53-F: 5′-GCTTTGAGGTGCGTGTTTGT-3′, R: 5′-TTGGGCAGTGCTCGCTTAG-3′; p21-F: 5′- ACCAGCATGACAGATTTCTACCA-3′, R: 5′-CAGAAGATGTAGAGCGGGCC-3′; PAI1-F: 5′-CTCATCAGCCACTGGAAAGGCA-3′, R: 5′-GACTCGTGAAGTCAGCCTGAAAC-3′; 14-3-3 F: 5′-TGCTGGACAGCCACCTCATCAA-3′, R: 5′-GGCTGAGTCAATGATGCGCTTC-3′; GADD45A-F: 5′-CTGGAGGAAGTGCTCAGCAAAG-3′, R: 5′-AGAGCCACATCTCTGTCGTCGT-3′.

### Cell migration assay

Cells (5 × 10^4^) were seeded into wells of the Oris Cell Migration Assembly Kit-FLEX (PlatypusTechnologies LLC, Fitchburg, WI, USA) and migration assays were conducted in accordance with the manufacturer’s instructions. After attached for 16 h, cells were allowed to migrate into the clear field after removal of the well inserts. Cells were then fixed with formaldehyde, stained with crystal violet and photographed. The pre-migration and post-migration images were analyzed using the ImageJ software (http://rsb.info.nih.gov/ij/).

Transwell assays were performed using Transwell inserts (Corning Costar) containing polycarbonate membrane filters (8-μm pore size) for 24-well plates. Cells (1 × 10^5^cells/well) in DMEM containing 1% FBS (100 μL) were plated into the upper chamber and DMEM containing 10% FBS (750 μL) was added to the lower chamber. After incubation at 37 °C under 5% CO2 for 22 h, cells on the upper side of the membrane were removed by cotton swabs, and cells on the bottom surface of the membrane were fixed in 75% methanol and 25% acetic acid for 15 min, stained with 0.5% crystal violet for 15 min, then washed 3 times with PBS. Five randomly selected fields were image captured with a dissecting microscope (Nikon SMZ1500) and the number of migration cells was counted.

### Statistical analysis

Quantitative data were presented as the mean ± SD, and the comparisons between groups were analyzed with a two-tailed, non parametric Student’s *t*-test. A p value of less than 0.05 was considered significantly different.

## Results

### Knockdown of *PRMT1* in a non-*MYCN*-amplified SK-N-SH neuroblastoma cells

**Figure 1:**
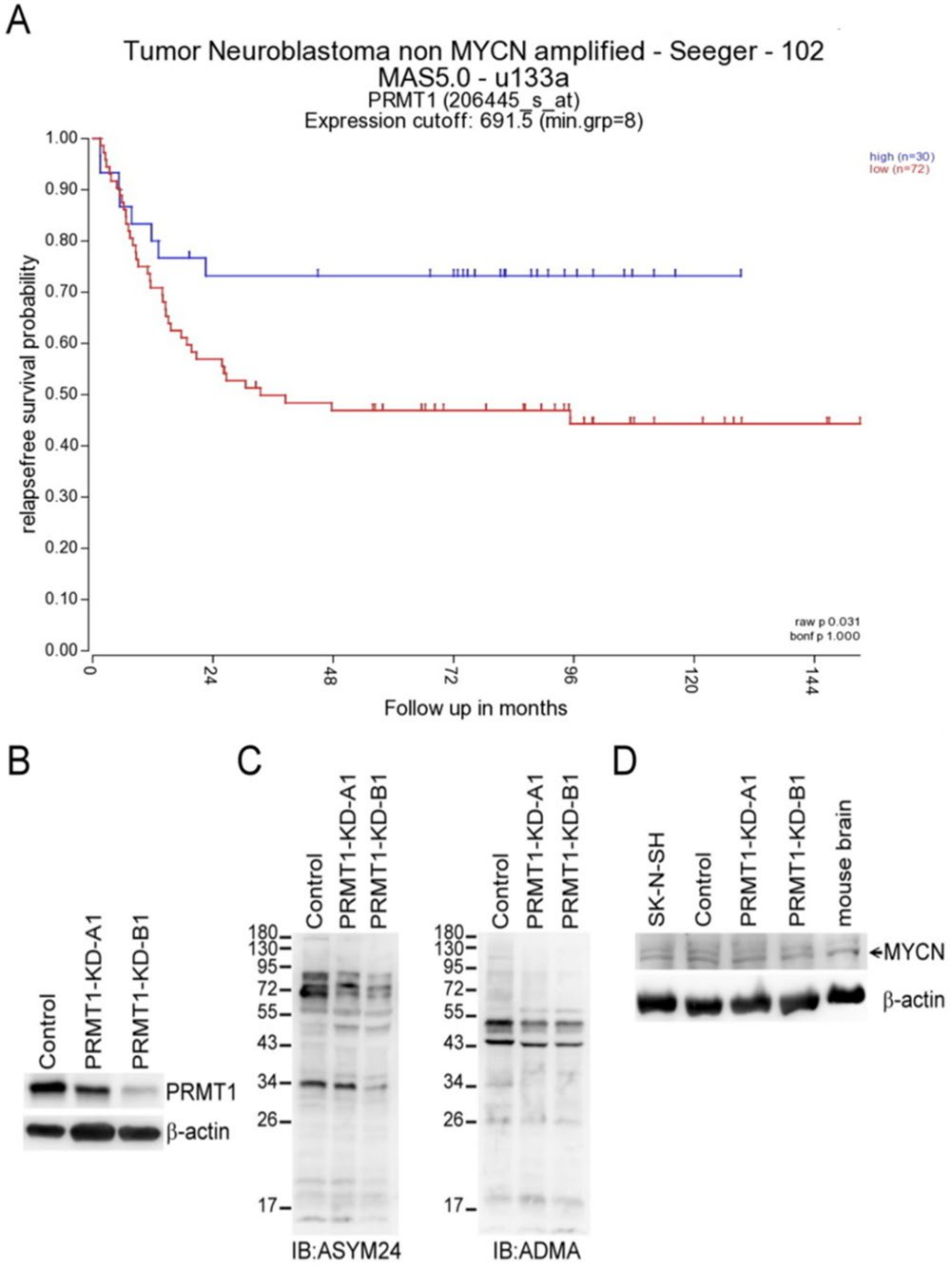
Association of low PRMT1 expression with poor prognosis in non-*MYCN*-amplified neuroblastoma patients and establishment of stable *PRMT1*-knockdown (*PRMT1*-KD) in a non-*MYCN*-amplified neuroblastoma cell line SK-N-SH. (**A**) Kaplan-Meier analysis of relapse-free survival for the Seeger dataset with 102 patients with non-*MYCN*-amplified neuroblastoma. The graph was downloaded from R2 genomics analysis and visualization platform (http://r2.amc.nl). The default “scan” modus was used to determine the cutoffof high and low expression. Patients with high PRMT1 expression were highlighted in blue, whereas patients with low PRMT1 expression were highlighted in red. (**B**) Cell extracts (20 μg of protein) from control vector-infected, *PRMT1* A1 or B1 shRNA-infected SK-N-SH cells were immunoblotted with anti-PRMT1. Detection by anti-β-actin was used as a loading control. (**C**) Cell extracts (20 μg of protein) were immunoblotted with asymmetric dimethylarginine-specific antibody ASYM24 (left) and ADMA (right). The immunoblots shown are the representatives of at least three independent experiments. (**D**) Extracts from non-infected, control vector-infected, *PRMT1* A1 or B1 shRNA-infected SK-N-SH cells, and mouse brain (50 μg of protein) were immunoblotted with anti-MYCN.

PRMT1 can be a direct target of *MYCN* in *MYCN*-amplified or overexpressed neuroblastoma^34^ and can modify and stabilize MYCN protein^35^. High PRMT1 expression levels had been reported to be strongly associated with poor prognostic outcomes of neuroblastoma^34,35^. However, the analyses of the Seeger dataset with 102 patients with non-*MYCN* amplified neuroblastoma using the R2 platform showed unfavorable prognosis in patients with low PRMT1 expression levels (Fig. 1A). The expression level of PRMT1 was not correlated with that of MYCN in these patients. Conversely, previous studies^34,35^ revealed that PRMT1 is positively correlated with MYCN in a large Kocak dataset with 476 patients with non-*MYCN* classified neuroblastoma (Supplementary Fig. 1).

We aimed to knock down *PRMT1* expression in a neuroblastoma cell line that is not *MYCN*-amplified or overexpressed to study the effects of low PRMT1 levels in these cells. We browsed the Expression Atlas with RNA sequencing data of 675 commonly used cancer cell lines (https://www.ebi.ac.uk/gxa/experiments/E-MTAB-2706/Results)^37^ and found that *MYCN* levels vary greatly in seven neuroblastoma cell lines included in the database, whereas *PRMT1* was expressed at a similar level (Supplementary Table S1). We used the SK-N-SH cell line with a low *MYCN* level in this study and knocked down the *PRMT1* expression via lentiviral shRNA infection. Successful stable knockdowns by either *PRMT1* A1 or B1 shRNA decreased the PRMT1 protein levels compared with that of non-infected or control shRNA-infected SK-N-SH cells (Fig. 1B). The reduced PRMT1 activity should greatly decrease the overall levels of ADMA-containing proteins in the PRMT1- knocked down (KD) cells because PRMT1 is the predominant type I PRMT responsible for the formation of asymmetric dimethylarginine (ADMA). We observed decreased levels of these signals in the *PRMT1*-KD cells by using ADMA-specific anti-ASYM24 and anti-ADMA antibodies that can detect different sets of ADMA-containing proteins^38,39^, (Fig. 1C). The expression levels of MYCN in the *PRMT1*-KD SK-N-SH cells and control cells were similar (Fig. 1D). Thus, we could further study the roles of PRMT1 independent of MYCN in these cells.

### Knockdown of *PRMT1* in SK-N-SH cells results in growth arrest and cellular senescence

**Figure 2:**
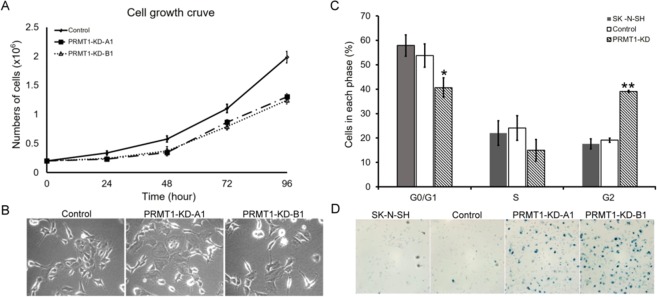
Senescent phenotypes and cell cycle arrest in *PRMT1*-KD SK-N-SH cells. (**A**) Doubling-time and growth curves of control and *PRMT1*-KD SK-N-SH cells. Cell growth curves of control, *PRMT1*-A1-KD and *PRMT1*-B1-KD cells determined by direct cell counts are shown. Data are the mean ± standard deviation (SD) of triplicates. (**B**) Cell morphology of non-infected, control vector-infected, *PRMT1* A1 or B1 shRNA-infected SK-N-SH cells. (**C**) Flow cytometry analyses of control or *PRMT1*-KD SK-N-SH cells with A1 and B1 shRNA. All experiments were performed at least three times. The data are shown as the mean ± SD. ^*^ indicates p < 0.05 and ^**^ indicates p < 0.01 compared with non-infected SK-N-SH cells. (**D**) Non-infected, control vector-infected, *PRMT1* A1 or B1 shRNA-infected SK-N-SH cells were fixed and stained for SA*-*β-Gal.

The stable *PRMT1*-KD SK-N-SH cells grew slowly with prolonged doubling time (control: 29.87 h, PRMT1-KD-A1: 34.77 h, PRMT1-KD-B1: 36.31 h) (Fig. 2A). Morphologically, *PRMT1*-KD cells were flattened and enlarged, and presented a number of short protrusions with small bulbs at the ends on the cell surface. The phenotypes were typical of the *PRMT1*-KD cells and were not detected in the control shRNA-infected or non-infected SK-N-SH cells (Fig. 2B).

We then analyzed the cell cycle of the *PRMT1*-KD SK-N-SH cells by flow cytometry. In Fig. 2C, more than half of the original and control vector-infected SK-N-SH cells were in the G_1_/G_0_ phase and less than 20% of the cells were in the G_2_/M phase. By contrast, more *PRMT1*-KD cells accumulated in the G_2_/M phase than in the G_1_/G_0_ phase.

The morphological characteristics of *PRMT1*-KD SK-N-SH cells were consistent with those of senescent phenotypes. Senescence-associated beta-galactosidase (SA-β-gal) is a widely used biomarker of cell senescence. Strong blue signals stained by SA-β-gal were detected in *PRMT1*-KD SK-N-SH cells but not in the control shRNA-infected or non-infected SK-N-SH cells (Fig. 2D). The results confirmed the cellular senescence in the neuroblastoma cells after the *PRMT1* expression was knocked down.

### Knockdown of *PRMT1* in SK-N-SH neuroblastoma cells increased p53 and p53-target genes expression at both RNA and protein levels

**Figure 3:**
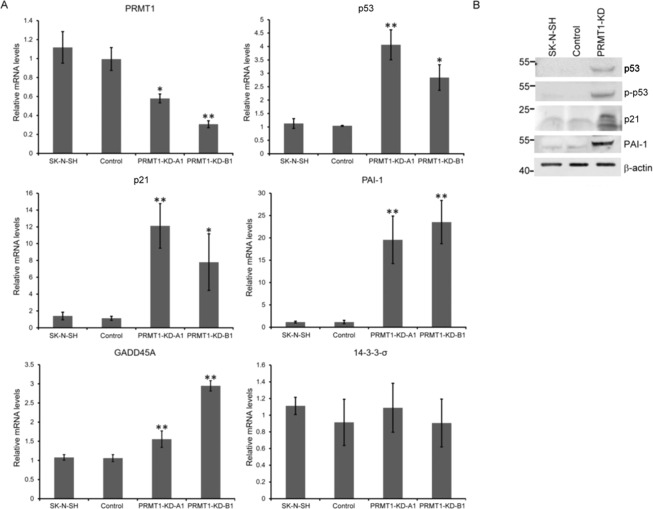
Increased expression of p53 and p53 targets in *PRMT1*-KD SK-N-SH cells. (**A**) RNA levels of *PRMT1*, *p5*3 and *p53* target *p21*, *PAI-1*, *GADD45* and *14-3-3-σ* in non-infected, control vector-infected, and PRMT1-A1 or B1 shRNA-infected SK-N-SH cells determined by qRT-PCR. Experiments were performed at least three times. The data are shown as the mean ± SD of triplicates. ^*^ indicates p < 0.05 and ^**^ indicates p < 0.01. (**B**) Western blot analyses of p53 and phosphorylated p53, p21 and PAI-1 in non-infected, control vector-infected, and *PRMT1* A1 plus B1 shRNA-infected (PRMT1-KD) SK-N-SH cells. The immunoblots shown are the representative of three independent experiments.

We then analyzed the genes that might link PRMT1 knockdown to the cellular senescence in the SK-N-SH cells. Considering that p53 is a key regulator for cellular senescence and cell cycle, we determined its expression level in *PRMT1*-KD and control SK-N-SH cells. qRT-PCR analyses showed a 2.6-fold to 4-fold increase in p53 mRNA levels in the *PRMT1*-KD cells compared with that in the control infected cells (Fig. 3A). Similarly, Western blot analyses revealed that the protein levels of p53 increased (Fig. 3B). The level of phosphorylated p53 that is responsible for transactivation also increased considerably in the *PRMT1*-KD cells (Fig. 3B). We then analyzed the expression of p53 targets that have been implicated to be critical in senescence. Both *p21* (cyclin-dependent kinase inhibitor 1 A) and *PAI-1* (*SERPINE1*) were highly induced by 10-fold to 20-fold (Fig. 3A). The levels of these proteins also increased (Fig. 3B). On the other hand, the mRNA level of one other p53 target gene *GADD45* only increased slightly and the level of another target *14-3-3-σ* did not increase in the *PRMT1*-KD SK-N-SH cells (Fig. 3A).

### DNA damage increased in *PRMT1*-KD SK-N-SH cells

**Figure 4:**
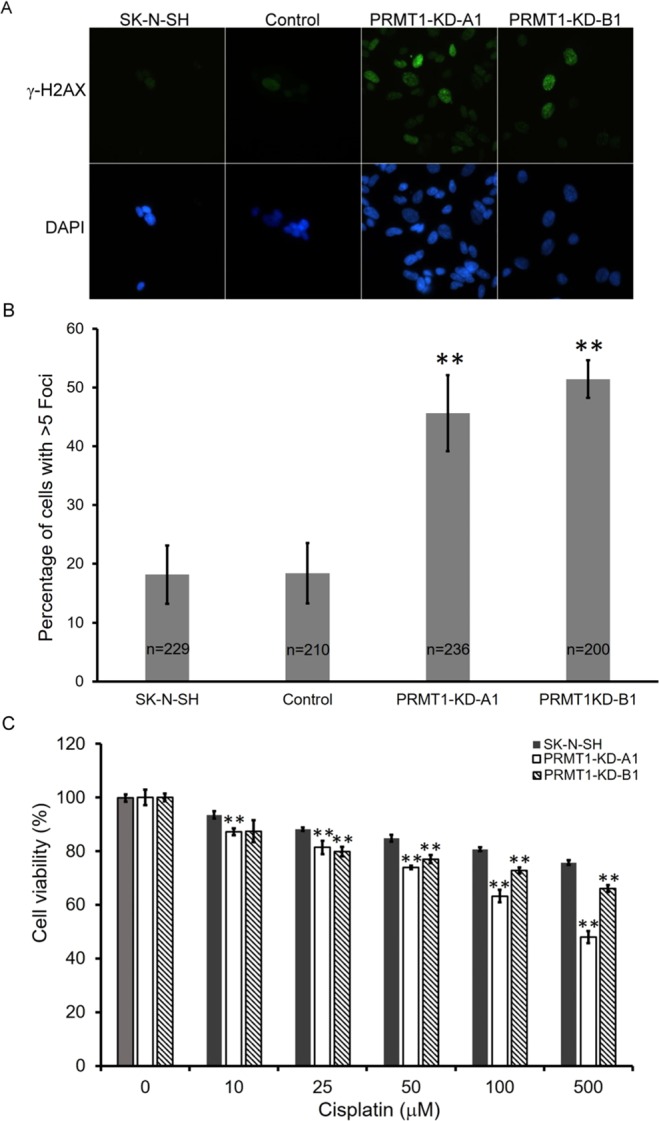
Increased DNA damage in *PRMT1*-KD SK-N-SH cells. (**A**) Immunoflourescent staining of phosphorylated H2AX (gamma-H2AX) in non-infected, control vector-infected and *PRMT1*A1 or B1 shRNA-infected SK-N-SH cells. (**B**) The cells with >5 foci of the gamma-H2AX staining were counted. Total number of non-infected, control vector-infected and *PRMT1*A1 or B1 shRNA-infected SK-N-SH cells counted were indicated in the graph. The statistical significance was assessed using the Student *t* test. (**C**) Cell viability of non-infected and *PRMT1* A1 or B1 shRNA-infected SK-N-SH cells with cisplatin treatment at various concentration for 24 h. Three independent experiments were performed. Data are shown as the mean ± SD of triplicates. ^**^ indicates p < 0.01 compared with non-infected SK-N-SH cells.

DNA damage is known to induce p53 expression. We examined whether the DNA damage levels increased in the *PRMT1*-KD SK-N-SH cells. The histone variant H2AX is phosphorylated in DNA damage response (DDR) and is widely used as an indicator of DNA damage. In Fig. 4A, the level of the phosphorylated H2AX (γ-H2AX) increased significantly in the *PRMT1*-KD cells but not in the control cells. We counted the number of γ-H2AX foci in the cells and observed that about 50% of the *PRMT1*-KD cells compared with ∼20% of the uninfected or control vector-infected cells had more than five foci (Fig. 4B). The fragmented DAPI staining indicated that the damaged DNA could be detected more frequently in the *PRMT1*-KD cells than in the control cells.

We also treated the cells with cisplatin, a chemotherapeutic agent that attacks rapidly dividing cancer cells via modifying DNA, thereby forming intra-strand cross links and other DNA lesions. The slightly reduced survival of *PRMT1*-KD cells treated with cisplatin was detected in comparison with that of the control cells (Fig. 4C).

### ROS production and sensitivity of the *PRMT1*-KD SK-N-SH cells

**Figure 5:**
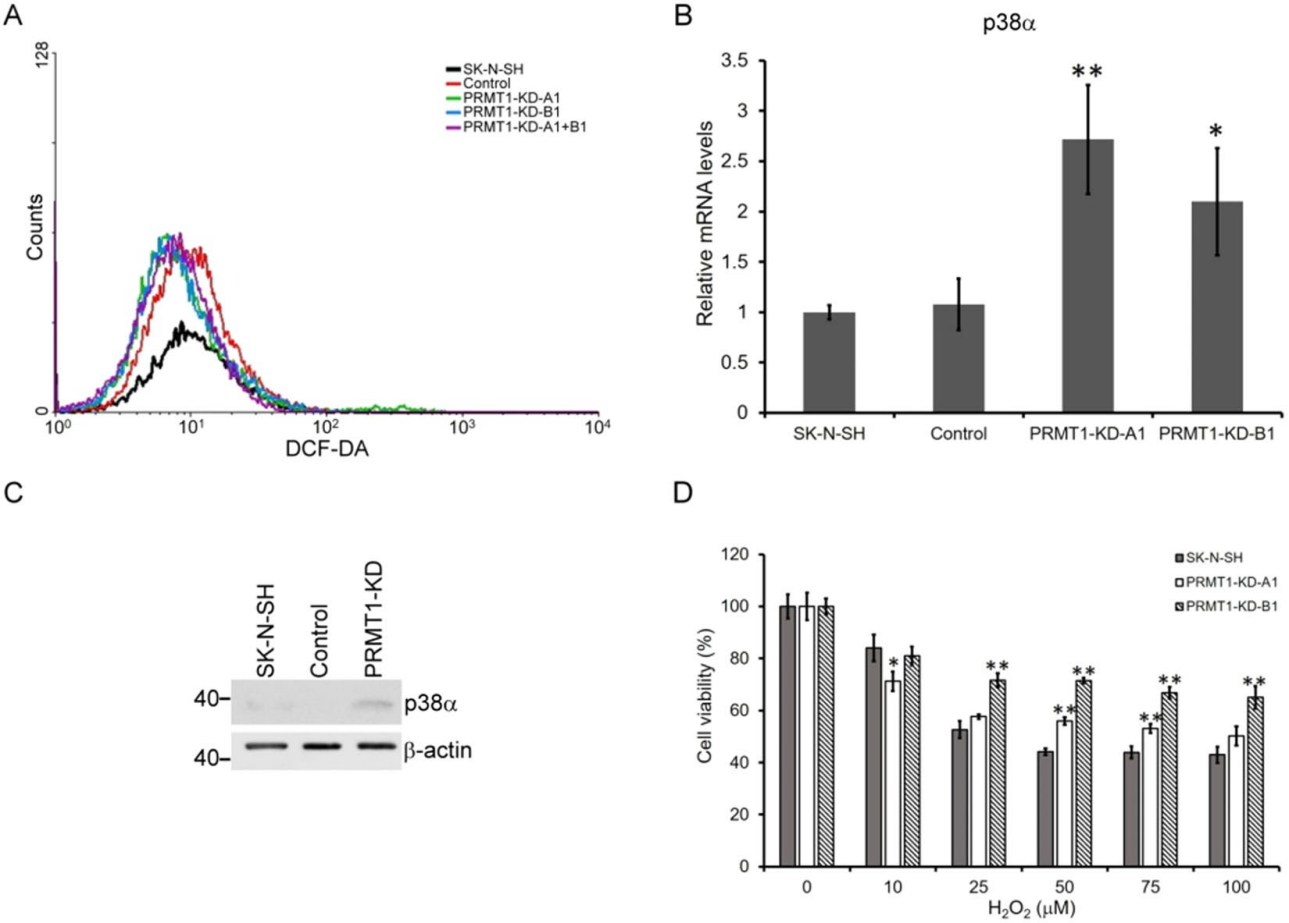
ROS production, sensitivity and signaling in *PRMT1*-KD SK-N-SH cells. (**A**) ROS production in non-infected, control vector-infected and *PRMT1* A1 or B1 shRNA-infected SK-N-SH cells as shown by the produced DCF-DA. (**B**) RNA levels of p38 in non-infected, control vector-infected, and *PRMT1*-A1 or B1 shRNA-infected KD SK-N-SH cells determined by RT-qPCR. (**C**) Western blot of p38 in non-infected, control vector-infected, and *PRMT1* A1 and B1 shRNA-infected SK-N-SH cells (100 μg of protein). (**D**) Survival of cells after addition of H_2_O_2_. Experiments were performed at least three times. The data are shown as the mean ± SD of triplicates. ^*^ indicates p < 0.05 and ^**^ indicates p < 0.01 compared with non-infected SK-N-SH cells.

One of the leading causes of replicative senescence is oxidative stress, and reactive oxygen species (ROS) can induce senescence. We then examined if the *PRMT1*-KD SK-N-SH cells might produce more ROS by measuring the staining with DFC-DA. The amount of ROS produced by the *PRMT1*-KD cells was slightly higher than that produced by the control cells (Fig. 5A). p38 MAPK plays important roles in ROS signaling. We observed p38α expression increased at both RNA and protein levels in the *PRMT1*-KD SK-N-SH cells (Fig. 5B,C), indicating that the ROS signaling pathway was activated. We then challenged the cells with hydrogen peroxide to examine their sensitivity to ROS. When the H_2_O_2_ concentration was 50 μM and higher, less than 50% of the normal SK-N-SH cells were viable. The number of *PRMT1*-KD cells observed upon hydrogen peroxide treatment was higher than that of the wild-type SK-N-SH cells, suggesting that they were more resistant to the oxidative stress (Fig. 5D).

### Increased cell migration in *PRMT1*-KD SK-N-SH cells can be suppressed with PAI-1 inhibitors

**Figure 6:**
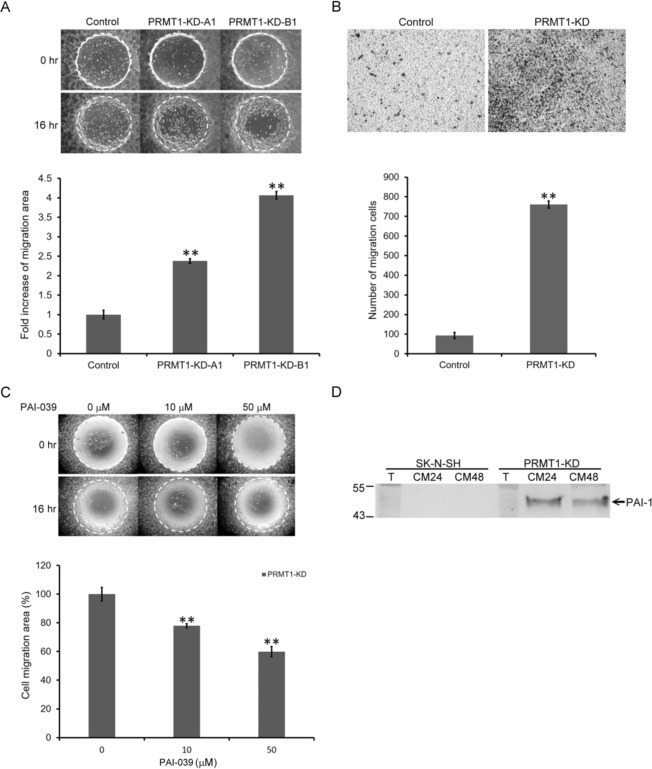
Migration activity of SK-N-SH cells increased after *PRMT1-*KD through PAI-1 induction. (**A**) The pre-migration (0 h) and post-migration (16 h) images of control vector-infected, *PRMT1* A1 or B1 shRNA-infected SK-N-SH cells are shown. The white circles indicate areas covered by the stoppers before cell migration. The area inside the white circle covered by cells is the migration area. Migration activity of the cells were quantified as the percentage of the migration area of the PRMT1 shRNA-infected cells compared to that of the control vector-uninfected SK-N-SH cells (^**^P < 0.01). (**B**) The cells that migrated to the bottom surface of the membrane after 22 h of Transwell migration assay were fixed and stained with crystal violet. Representative images of *PRMT1* A1 shRNA-infected or control vector-infected SK-N-SH cells were shown. Five randomly selected fields were imaged and the number of the migrated cells was counted and data are shown as the mean ± SD. ^**^ indicates p < 0.01 compared with non-infected SK-N-SH cells. (**C**) The pre-migration (0 h) and post-migration (16 h) images of *PRMT1* B1 shRNA-infected SK-N-SH cells treated with a PAI-1 inhibitor PAI-039 at the concentration of 0, 10, and 50 μM. Migration activity were quantified as the percentage of the migration area of the treated cells over untreated cells (^**^P < 0.01). (**D**) Western blot analyses of PAI-1 in the spent media of non-infected and *PRMT1*-A1 shRNA-infected SK-N-SH cells. T indicates total lysate. CM24 and CM48 indicate serum free culture medium from 24 h and 48 h cultures concentrated by ultracentrifugation.

The increased expression and secretion of PAI-1 are critical for replicative senescence downstream of p53 in cells^40^. PAI-1 is related to cell migration activities through the suppression of plasminogen activator (PA). We analyzed if the *PRMT1*-KD cells with elevated PAI-1 levels might show different migration activities. Though control SK-N-SH cells barely migrated, *PRMT1*-KD cells exhibited highly detectable migration with increased migration areas (Fig. 6A). We further conducted the Transwell experiments to confirm the increased migration activity of the *PRMT1*-KD cells. The results showed that significantly more *PRMT1*-KD cells passed through the membrane compared to control cells (Fig. 6B).

We then treated the cells with a PAI-1 inhibitor PAI-039. At the concentration that did not affect cell survival, PAI-039 treatment decreased the migration of *PRMT1*-KD cells in a dose-dependent manner (Fig. 6C). Normal SK-N-SH cells showed a limited migration activity regardless of the inhibitor treatment (data not shown). We also collected and analyzed the spent media, and our results validated that the secretion of PAI-1 increased in the *PRMT1*-KD SK-N-SH cells (Fig. 6D).

### Induced p21/PAI-1 expression in *PRMT1*-KD SK-N-SH cells requires p53

**Figure 7:**
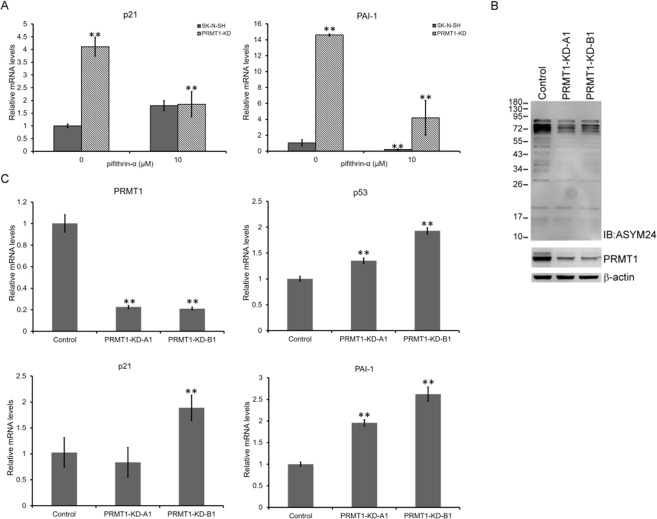
The requirement of p53 to induce p21 and PAI-1 in non-*MYCN*-amplified neuroblastoma cells. (**A**) The SK-N-SH cells infected with *PRMT1 *A1 plus B1 shRNA at the fifth day were treated with a p53 inhibitor pifithrin-α (10 μM) or not for 48 h. The RNA levels of the *p53* target *p21* and *PAI-1* of the non-infected and *PRMT1*-KD SK-N-SH cells treated with pifithrin-α or not were determined. (**B**) Western blot analyses of cell extracts (20 μg of protein) from control vector infected, *PRMT1* A1 or B1 shRNA-infected SK-N-AS cells with anti-PRMT1 or ASYM24. Detection by anti-β-actin was used as a loading control. (**C**) RT-qPCR analyses of RNA levels of *PRMT1*, *p53*, *p21* and *PAI-1* in non-infected, control vector-infected, and *PRMT1 *A1 or B1 shRNA-infected SK-N-AS cells. Experiments were performed at least three times. The data are shown as the mean ± SD of triplicates. ^**^ indicates p < 0.01.

To further examine if the induction of p21 and PAI-1 after *PRMT1*-KD is p53-dependent, we used a p53 transactivation inhibitor pifithrin-α to treat thenewly infected SK-N-SH cells. The induced levels of p21 and PAI-1 in *PRMT1*-KD SK-N-SH cells declined after the cells were incubated with the p53 inhibitor (Fig. 7A). This finding supportsan upstream role for p53.

The critical role of p53 in the induction of p21 and PAI-1 was further illustrated in SK-N-AS, another non-MYCN-overexpressing neuroblastoma cell line (Supplementary table) whose p53 is not functional^41^. The levels of PRMT1 as well as those of asymmetric dimethylarginine containing proteins decreased when PRMT1 was knocked down by *PRMT1* A1 or B1 shRNA in SK-N-AS cells (Fig. 7B). Different from the flattened and enlarged cell morphology observed in the *PRMT1*-KD SK-N-SH cells (Fig. 2B), no distinctive senescent phenotypes could be observed in the stable *PRMT1*-KD SK-N-AS cells (Supplementary Fig. 2). In comparison with ~10-fold to 20-fold of p21 and PAI-1 induction in *PRMT1*-KD SK-N-SH cells (Fig. 3A), low induction levels (<2.5-fold) were detected in the *PRMT1* shRNA*-*infected SK-N-AS cells after two weeks of selection (Fig. 7C).

### Inhibition of PRMT1 induced p21 and PAI-1 in SK-N-SH and other cell lines

**Figure 8:**
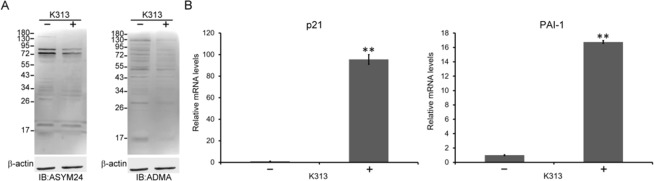
Inhibition of PRMT1 by a specific inhibitor K313 induced p21 and PAI-1 expression in SK-N-SH cells. (**A**) SK-N-SH cells were treated with a PRMT1 inhibitor K313 (2 μM) or not for 7 days with medium change every 3 days. Cell extracts (25 μg of protein) were immunoblotted with asymmetric di-methylarginine-specific antibodies ASYM24 (left) and ADMA (right). +/−  indicate the addition of K313 or not. The immunoblots shown are representatives of independent experiments. (**B**) RNA levels of p53 target p21 and PAI-1 under the treatment of K313 or not. ^**^ indicates p < 0.01.

The induced p21 and PAI-1 expression might be critical for the phenotypes of the *PRMT1*-KD SK-N-SH cells. To confirm whether the induction was related to PRMT1 suppression, we treated the SK-N-SH cells with a newly developed PRMT1-specific inhibitor K313, in addition to the shRNA knockdown of PRMT1. The selectivity panel screen exhibited the specific inhibitory effects of K313 on PRMT1 activity in comparison with other PRMTs (Supplementary Fig. 3). K313 effectively reduced the ADMA-containing proteins in SK-N-SH cells at the concentration of 2 μM (Fig. 8A). Short-term (3 days) K313 treatment had no effects on the senescence genes (data not shown), whereas the expression levels of p21 and PAI-1 increased after 7 day of treatment (Fig. 8B). These results are in agreement with those of the PRMT1 knockdown experiments.

To examine the effect of low PRMT1 levels in other non-*MYCN* overexpressed cells, we used SK-N-MC, another cell line established and characterized in the same study with SK-N-SH, but which was derived from a different patient with neuroblastoma^42^. Considering that SK-N-MC cells were sensitive to lentiviral infection and that no stable *PRMT1*-KD or control shRNA-infected cell lines could be established, we treated SK-N-MC cells with K313 to evaluate the effects of low PRMT1 activities in this cell line. K313 treatment decreased the ADMA levels in SK-N-MC cells. The cells showed senescent phenotypes and increased the expression of *p53*, *p21* and *PAI-1* after K313 treatment for 6 days (Supplementary Fig. 4). These results were consistent with those observed in SK-N-SH cells.

We had conducted PRMT1 studies in HeLa cells and in an oral cancer cell line SAS^31,43^. In the present study, we thus conducted similar knockdown experiments on HeLa and SAS cells to determine if senescent phenotypes might occur. SA-β-gal staining was enhanced in *PRMT1*-KD HeLa cells (Supplementary Fig. 5 A), but to a lower level than in SK-N-SH cells. PRMT1 knockdown in HeLa cells slightly increased the expression levels of p53 but not that of p21 and PAI-1 (Supplementary Fig. 5B). Though we had reported decreased growth rates in *PRMT1*-KD SAS cells^31^, these cells did not show senescence or p53, p21, and PAI-1 induction after PRMT1 knockdown (Supplementary Fig. 5C,D).

## Discussion

PRMT1 is the predominant type I protein arginine methyltransferase that catalyzes the methylation of numerous substrate proteins involved in various cellular pathways. PRMT1 can act as a co-activator in the epigenetic process^5,6^ and is involved in tumorigenesis. High PRMT1 expression levels are correlated with poor prognosis of neuroblastoma^34^. The amplification or overexpression of *MYCN* represent a major genetic defect and accounts for the poor prognosis of neuroblastoma. *PRMT1* is among the *MYCN* signature target genes and modification by PRMT1 can stabilize MYCN. These studies suggested that PRMT1 can be a promising target for MYCN-driven neuroblastoma^35^. However, we found that the PRMT1 expression levels were not correlated with MYCN levels in a cohort of patients with non-*MYCN*-amplified neuroblastoma (Supplementary Fig. 1). In this cohort, the patients with low PRMT1 levels showed poor prognostic outcomes (Fig. 1A). Thus, the roles of PRMT1 in patients with non-*MYCN*-amplified neuroblastoma should be further investigated.

In this study, we used the non-*MYCN*-amplified or overexpressed neuroblastoma cell line SK-N-SH. *PRMT1*-KD SK-N-SH cells proliferate slowly, an observation that is similar to that of the *MYCN*-overexpressed neuroblastoma cells with PRMT1 knockdown^35^. We also observed cellular senescence in the *PRMT1*-KD SK-N-SH cells (Fig. 2) that was previously not reported in neuroblastoma cells. The downregulation of PRMT1 results in reduced cell growth and cell cycle arrest in a spectrum of cancer cell lines^30,31,44–46^. For example, when PRMT1 was knocked down in glioma cells, the cells were arrested in G_1_/S with increased apoptosis^47^. In MDA-MB-231 breast cancer cell line, the knockdown of PRMT1 accumulates cells in the G_2_/M phase and results in senescence^48^. Similarly, we detected cell cycle arrest in the G_2_/M phase and senescence.

We observed p53 induction at the RNA and protein levels in the *PRMT1*-KD SK-N-SH cells (Fig. 3). The phosphorylation of p53 also increased, indication that transactivation occurred. The expression of p21, a p53 target and cyclin-dependent kinase inhibitor critical for cell senescence, was strongly induced. PAI-1, another p53 downstream senescent factor, was also induced to a high level (Fig. 3). Suppressing p53 transactivity repressed the induction of p21 and PAI-1 in the newly established *PRMT1*-KD SK-N-SH cells. Moreover, *PRMT1* KD in a p53-null non-*MYCN*-amplified neuroblastoma SK-N-AS cells could barely induce p21/PAI-1 (Fig. 7). These results support critical roles for p53 in inducing the senescence genes in the non-*MYCN*-amplified neuroblastoma cells with *PRMT1* KD.

Consistent with cellular senescence, we observed increased DNA damage in the *PRMT1*-KD SK-N-SH cells (Fig. 4). PRMT1 knockout in the MEF cells led to spontaneous DNA damage^20^. We detected increased γ-H2AX levels in the *PRMT1*-KD SK-N-SH cells, indicating that DNA damage is exacerbated. These cells were also more sensitive to cisplatin, a chemotherapeutic chemical that cross-links DNA (Fig. 4). Besides, we showed that the ROS production in the *PRMT1*-KD SK-N-SH cells marginally increased (Fig. 5). The higher intracellular ROS levels activated ROS signaling as shown by p38 induction. Neuroblastoma cells, such as SK-N-SH, have been used to evaluate the neurotoxicity of ROS in neural degenerative diseases because of their neuronal origin^49^. Hydrogen peroxide-induced cytotoxicity in neuroblastoma cells has also been considered in developing therapeutic agents^50^. The *PRMT1*-KD SK-N-SH cells may adapt to the increased ROS levels as they were slightly more resistant to H_2_O_2_. The ROS levels in the *PRMT1*-KD SK-N-SH cells may reach the threshold and induced a protective hormetic response^51^. Nevertheless, the ROS accumulation may further contribute to tumor progression by increasing chromosome instability^52^.

PAI-1 is synthesized and secreted in senescent cells^53^. It is a critical downstream target of p53 and is necessary and sufficient to induce senescence^40^. Senescence is a special state of durable cell cycle arrest and can contribute to cancer prevention. Nevertheless, senescent cells may develop a senescence-associated secretory phenotype (SASP)^54^ by secreting SASP factors, such as cytokines and growth factors to create a microenvironment for tumorigenesis. PAI-1 and the serine proteases, namely urokinase- or tissue-type plasminogen activator, are also SASP factors^54^. In this study, PAI-1 was highly induced as well as secreted in the *PRMT1-*KD cells upon senescence (Figs 2, 6). PAI -1 induction was dependent on p53 (Fig. 3), and treatment with a *PRMT1*-specific inhibitor K313 also induced PAI-1 (Fig. 8), suggesting that a low functional PRMT1 activity derepressed the p53-PAI-1 expression in the SK-N-SH cells. To our knowledge, this is the first report demonstrating a PRMT1-p53-PAI-1 relationship in PRMT1 studies.

Considering that PAI-1 is critical in tumor migration and invasion, we examined the migration activity of the stable *PRMT1*-KD SK-N-SH cells. The uninfected SK-N-SH cells or the control vector-infected cells showed low migration activities. The *PRMT1* knockdown significantly increased the migration activity of the SK-N-SH cells in different migration assays (Fig. 6). However, we found opposite positive correlations between PRMT1 and the cell mobility compared to previous studies. Specifically, *prmt1* knockdown by antisense morpholino injection greatly reduces the mobility of the cells in zebrafish embryos, and *PRMT1* knockdown by siRNA in a hepatoma cell line Huh-7 decreases migration activities^55^. Similarly, *prmt1* knockdown by shRNA in an oral cancer cell line SAS represses migration^31^. Sugiura *et al*. did not detect PAI-1 expression in neuroblastoma tumor cells, but found that PAI-1 expression mainly by endothelial cells in the surrounding stroma promotes metastasis^55^. Consistently, we could barely detect PAI-1 expression in the original SK-N-SH cells through RT-PCR. The treatment with the PAI-1 inhibitor in the *PRMT1*-KD SK-N-SH cells decreased the migration activity (Fig. 6), indicating that the induced PAI-1 at least partially accounted for the increased migration activity. Therefore, the increased mobility of the SK-N-SH neuroblastoma cells after PRMT1 knockdown might be specific to the cell line and associated with the significantly increased PAI-1 levels. The results also indicate the possibility that the PAI-1 induction and secretion observed in senescent neuroblastoma cells may promote metastasis of neighboring tumor cells.

We continuously observed cellular senescence and p53/p21/PAI-1 induction in different batches of stable *PRMT1*-KD SK-N-SH cells. A long incubation (6-7 days) but not short-term (2–3 days) treatment with the PRMT1 inhibitor K313 could induce p21/PAI-1 in SK-N-SH and another non-MYCN overexpressed SK-N-MC cells. The SK-N-MC cells could not endure long-term selection after lentiviral infection and no stable cell lines could be established for senescent studies. Transient infection could suppressthe PRMT1 expression, but could not induce senescent phenotypes or genes (data not shown). Thus, the cellular senescence induced by PRMT1 knockdown and the effects on p53-p21/PAI expression in non-*MYCN*-amplified neuroblastoma cells might only be evident in a specific time window after the long-term repression of PRMT1. We suspect that DNA damage that accumulate through a period of time might induce not only *p53* and but also p21 and PAI-1 in the *PRMT1-*KD SK-N-SH cells. However, the long-term suppression of PRMT1 might result in compound effects and lead to context-dependent phenotypes in different cells because PRMT1 is a protein arginine methyltransferase catalyzing the modification of numerous substrate proteins involved in various cellular processes.

By comparison, *PRMT1*-KD in oral cancer SAS cells did not induce detectable cellular senescence nor p53 induction in these rapidly growing cells^31^. *PRMT1*-KD in HeLa cells enhanced the SA-β-gal staining and increased the expression level of p53 marginally. Neither cell line induced p21/PAI-1 after *PRMT1*-KD (Supplementary Fig. 5), in agreement with the induction of p53 and p21/PAI-1 by *PRMT1* knockdown being cell-type specific.

A negative expression correlation between PRMT1 and p53 could not be detected in the non-*MYCN* amplified Seeger cohort with 102 patients. Further studies should determine whether this finding is due to the limited sample size. Interestingly, we observed the opposite positive correlations between *PRMT1* and *p53* in the Kocak dataset with 649 patients with non-discriminated neuroblastoma (Supplementary Fig. 6). There was no correlation between *PRMT1* and *p21 *or *PAI-1* in either dataset (data not shown). Nevertheless, our findings are consistent with those in cell line studies, suggesting a PRMT1-p53-p21/PAI connection in a neuroblastoma subgroup under specific conditions.

Previous screening identified PRMT1 as a crucial target for the vulnerability of p53-null osteosarcomas^56^. PRMT1 functions as a coactivator of p53 and works independently or cooperatively with p300 and CARM1 in mediating activation of GADD45 by p53^57^. Interestingly, our results indicated a new repressive effect of PRMT1 on the modulation of p53 and downstream p21/PAI-1 expression. The results could be compared with the growth arrest and senescence by PRMT6 deficiency reported in several studies^58–60^. PRMT6 is another type I PRMT family member responsive for the repressive epigenetic ADMA marks on H3R2 (H3R2me2a). PRMT6 and H3R2me2a bind to the promoter regions of *p53* in MEF, with subsequent activation of *p21* but not *PAI-1*^58^; it may also directly bind to *p21* in a *p53*-independent way in various cancer cells to negatively regulate the transcription^59,60^. Nevertheless, previous experiments showed that PRMT1 is not associated with the *p53* promoter and is less likely to be a direct transcriptional corepressor as PRMT6^58^.

In conclusion, even if *PRMT1*-KD SK-N-SH cells grew slowly and became senescent, the cells survived and gained increased migration abilities. While senescence and reduced cell cycle progression are often associated with tumor suppression, significantly elevated PAI-1 level due to p53 induction might lead to adverse tumor progression. Somatic mutations in *p53* are frequently encountered in human tumors. Nevertheless, the *p53* gene in neuroblastoma at diagnosis is rarely mutated^41^. As such, low levels of PRMT1 should be able to induce p21/PAI-1 as shown in SK-N-SH cells through the wild-type p53. The whole picture of PRMT1 in neuroblastoma cells without MYCN overexpression is likely to be much more complicated than what we showed in the SK-N-SH cells. Our data may partially explain clinical data showing that low PRMT1 expression is a poor prognostic factor in patients with non-*MYCN*-amplified neuroblastoma. Thus the multifaceted effects of PRMT1 should be carefully considered when contemplating PRMT1 as the therapeutic target.

## Supplementary information

Supplementary Figure

## References

[1] Bedford MT, Clarke SG (2009). Protein Arginine Methylation in Mammals: Who, What, and Why. Molecular Cell.

[2] Blanc RS, Richard S (2017). Arginine Methylation: The Coming of Age. Mol Cell.

[3] Lin WJ, Gary JD, Yang MC, Clarke S, Herschman HR (1996). The mammalian immediate-early TIS21 protein and the leukemia-associated BTG1 protein interact with a protein-arginine N-methyltransferase. J Biol Chem.

[4] Tang J (2000). PRMT1 is the predominant type I protein arginine methyltransferase in mammalian cells. J Biol Chem.

[5] Wang Y-C, Li C (2012). Evolutionarily conserved protein arginine methyltransferases in non-mammalian animal systems. FEBS Journal.

[6] Nicholson TB, Chen T, Richard S (2009). The physiological and pathophysiological role of PRMT1-mediated protein arginine methylation. Pharmacological Research.

[7] Pahlich S, Zakaryan RP, Gehring H (2006). Protein arginine methylation: Cellular functions and methods of analysis. Biochimica et Biophysica Acta (BBA) - Proteins & Proteomics.

[8] Abramovich C, Yakobson B, Chebath J, Revel M (1997). A protein-arginine methyltransferase binds to the intracytoplasmic domain of the IFNAR1 chain in the type I interferon receptor. EMBO J.

[9] Mowen KA (2001). Arginine methylation of STAT1 modulates IFNalpha/beta-induced transcription. Cell.

[10] Weber S (2009). PRMT1-mediated arginine methylation of PIAS1 regulates STAT1 signaling. Genes & Development.

[11] Blanchet F, Schurter BT, Acuto O (2006). Protein arginine methylation in lymphocyte signaling. Current Opinion in Immunology.

[12] Reintjes A (2016). Asymmetric arginine dimethylation of RelA provides a repressive mark to modulate TNFalpha/NF-kappaB response. Proc Natl Acad Sci USA.

[13] Yamagata K (2008). Arginine methylation of FOXO transcription factors inhibits their phosphorylation by Akt. Mol Cell.

[14] Wang H (2001). Methylation of histone H4 at arginine 3 facilitating transcriptional activation by nuclear hormone receptor. Science.

[15] Stallcup MR (2000). Co-operation between protein-acetylating and protein-methylating co-activators in transcriptional activation. Biochem Soc Trans.

[16] Boisvert FM, Dery U, Masson JY, Richard S (2005). Arginine methylation of MRE11 by PRMT1 is required for DNA damage checkpoint control. Genes Dev.

[17] Boisvert FM, Rhie A, Richard S, Doherty AJ (2005). The GAR motif of 53BP1 is arginine methylated by PRMT1 and is necessary for 53BP1 DNA binding activity. Cell Cycle.

[18] Dery U (2008). A glycine-arginine domain in control of the human MRE11 DNA repair protein. Mol Cell Biol.

[19] Pawlak MR, Scherer CA, Chen J, Roshon MJ, Ruley HE (2000). Arginine N-methyltransferase 1 is required for early postimplantation mouse development, but cells deficient in the enzyme are viable. Mol Cell Biol2.

[20] Yu Z, Chen T, Hebert J, Li E, Richard S (2009). A Mouse PRMT1 Null Allele Defines an Essential Role for Arginine Methylation in Genome Maintenance andCell Proliferation. Molecular and Cellular Biology.

[21] Tsai YJ (2011). The predominant protein arginine methyltransferase PRMT1 is critical for zebrafish convergence and extension during gastrulation. FEBS J.

[22] Hashimoto M (2016). Severe Hypomyelination and Developmental Defects Are Caused in Mice Lacking Protein Arginine Methyltransferase 1 (PRMT1) in the Central Nervous System. J Biol Chem.

[23] Wu TT (2010). Polymorphisms of the RET gene in hirschsprung disease, anorectal malformation and intestinal pseudo-obstruction in Taiwan. J Formos Med Assoc.

[24] Wu TT (2005). Low RET mutation frequency and polymorphism analysis of the RET and EDNRB genes in patients with Hirschsprung disease in Taiwan. J Hum Genet.

[25] Wu TT (2010). Analyses of PRMT1 proteins in human colon tissues from Hirschsprung disease patients. Neurogastroenterol Motil.

[26] Gallik, K. L. *et al*. Neural crest and cancer: Divergent travelers on similar paths. *Mechanisms of development*, 10.1016/j.mod.2017.08.002 (2017). 10.1016/j.mod.2017.08.002 PMC5811199 28888421

[27] Maris JM, Hogarty MD, Bagatell R, Cohn SL (2007). Neuroblastoma. Lancet (London, England).

[28] Yoshimatsu M (2011). Dysregulation of PRMT1 and PRMT 6, Type I arginine methyltransferases, is involved in various types of human cancers. Int J Cancer.

[29] Li B, Liu L, Li X, Wu L (2015). miR-503 suppresses metastasis of hepatocellular carcinoma cell by targeting PRMT1. Biochem Biophys Res Commun.

[30] Zhou, W., Yue, H., Li, C., Chen, H. & Yuan, Y. Protein arginine methyltransferase 1 promoted the growth and migration of cancer cells in esophageal squamous cell carcinoma. *Tumour Biol*, 10.1007/s13277-015-4098-3 (2015). 10.1007/s13277-015-4098-3 26392112

[31] Chuang CY (2017). PRMT1 expression is elevated in head and neck cancer and inhibition of protein arginine methylation by adenosine dialdehyde or PRMT1 knockdown downregulates proliferation and migration of oral cancer cells. Oncology reports.

[32] Cimato TR (2002). Nerve growth factor-mediated increases in protein methylation occur predominantly at type I arginine methylation sites and involve protein arginine methyltransferase 1. J Neurosci Res.

[33] Miyata S, Mori Y, Tohyama M (2008). PRMT1 and Btg2 regulates neurite outgrowth of Neuro2a cells. Neuroscience Letters.

[34] Valentijn LJ (2012). Functional MYCN signature predicts outcome of neuroblastoma irrespective of MYCN amplification. Proc Natl Acad Sci USA.

[35] Eberhardt, A. *et al*. Protein arginine methyltransferase 1 is a novel regulator of MYCN in neuroblastoma. *Oncotarget*, 10.18632/oncotarget.11556 (2016). 10.18632/oncotarget.11556 PMC5325390 27571165

[36] Rettig WJ, Spengler BA, Chesa PG, Old LJ, Biedler JL (1987). Coordinate changes in neuronal phenotype and surface antigen expression in human neuroblastoma cell variants. Cancer Res.

[37] Mandriota SJ (2015). Ataxia-telangiectasia mutated (ATM) silencing promotes neuroblastoma progression through a MYCN independent mechanism. Oncotarget.

[38] Boisvert FM (2003). A Proteomic Analysis of Arginine-methylated Protein Complexes. Molecular & Cellular Proteomics.

[39] Guo A (2014). Immunoaffinity enrichment and mass spectrometry analysis of protein methylation. Mol Cell Proteomics.

[40] Kortlever RM, Higgins PJ, Bernards R (2006). Plasminogen activator inhibitor-1 is a critical downstream target of p53 in the induction of replicative senescence. Nat Cell Biol.

[41] Goldschneider D (2006). Expression of C-terminal deleted p53 isoforms in neuroblastoma. Nucleic Acids Res.

[42] Biedler JL, Helson L, Spengler BA (1973). Morphology and growth, tumorigenicity, and cytogenetics of human neuroblastoma cells in continuous culture. Cancer Res.

[43] Lee YJ, Hsieh WY, Chen LY, Li C (2012). Protein arginine methylation of SERBP1 by protein arginine methyltransferase 1 affects cytoplasmic/nuclear distribution. J Cell Biochem.

[44] Akter, K. A. *et al*. FAM98A is a novel substrate of PRMT1 required for tumor cell migration, invasion, and colony formation. *Tumour Biol*, 10.1007/s13277-015-4310-5 (2015). 10.1007/s13277-015-4310-5 26503212

[45] Le Romancer M (2008). Regulation of Estrogen Rapid Signaling through Arginine Methylation by PRMT1. Molecular Cell.

[46] Gou Q, He S, Zhou Z (2017). Protein arginine N-methyltransferase 1 promotes the proliferation and metastasis of hepatocellular carcinoma cells. Tumour Biol.

[47] Wang S (2012). The role of protein arginine-methyltransferase 1 in gliomagenesis. BMB reports.

[48] Gao Y (2016). The dual function of PRMT1 in modulating epithelial-mesenchymal transition and cellular senescence in breast cancer cells through regulation of ZEB1. Scientific reports.

[49] Wu YL (2017). Treatment with Caffeic Acid and Resveratrol Alleviates Oxidative Stress Induced Neurotoxicity in Cell and Drosophila Models of Spinocerebellar Ataxia Type3. Scientific reports.

[50] Ma E (2017). Pharmacologic ascorbate induces neuroblastoma cell death by hydrogen peroxide mediated DNA damage and reduction in cancer cell glycolysis. Free radical biology & medicine.

[51] Schieber M, Chandel NS (2014). ROS function in redox signaling and oxidative stress. Curr Biol2.

[52] Wu Y, Antony S, Meitzler JL, Doroshow JH (2014). Molecular mechanisms underlying chronic inflammation-associated cancers. Cancer Lett.

[53] Kunz C, Pebler S, Otte J, von der Ahe D (1995). Differential regulation of plasminogen activator and inhibitor gene transcription by the tumor suppressorp53. Nucleic Acids Res.

[54] Coppe JP, Desprez PY, Krtolica A, Campisi J (2010). The senescence-associated secretory phenotype: the dark side of tumor suppression. Annual review of pathology.

[55] Sugiura Y (1999). The plasminogen-plasminogen activator (PA) system in neuroblastoma: role of PA inhibitor-1 in metastasis. Cancer Res5.

[56] Hsu JH (2017). PRMT1-Mediated Translation Regulation Is a Crucial Vulnerability of Cancer. Cancer Res7.

[57] An W, Kim J, Roeder RG (2004). Ordered Cooperative Functions of PRMT1, p300, and CARM1 in Transcriptional Activation by p53. Cell.

[58] Neault M, Mallette FA, Vogel G, Michaud-Levesque J, Richard S (2012). Ablation of PRMT 6 reveals a role as a negative transcriptional regulator of the p53 tumor suppressor. Nucleic Acids Res.

[59] Phalke S (2012). p53-Independent regulation of p21Waf1/Cip1 expression and senescence by PRMT 6. Nucleic Acids Res.

[60] Stein C, Riedl S, Ruthnick D, Notzold RR, Bauer UM (2012). The arginine methyltransferase PRMT 6 regulates cell proliferation and senescence through transcriptional repression of tumor suppressor genes. Nucleic Acids Res.

