# Supplementary material for: Downregulation of PRMT1 promotes the senescence and migration of a non-MYCN amplified neuroblastoma SK-N-SH cells
Source: Sci Rep. 2019 Feb 11;9:1771. doi: 10.1038/s41598-018-38394-6 (PMC6370813; doi:10.1038/s41598-018-38394-6)
Supplement: Supplementary file 1 — Supplementary Figure [file 41598_2018_38394_MOESM1_ESM.pdf]

Downregulation of PRMT1 promotes the senescence and migration of a non-*MYCN* amplified neuroblastoma SK-N-SH cells

Yu-Jen Lee[1,2], Wen-Wei Chang[1,3], Chien-Ping Chang[1], Tsung-Yun Liu[1], Chun-Yi Chuang[4,5], Kun Qian[6], Y. George Zheng[6], and Chuan Li[1,3,*]

[1]Department of Biomedical Sciences, Chung Shan Medical University, Taichung, Taiwan; [2]Department of Medical Research, Jen-Ai Hospital, Taichung, Taiwan; [3]Department of Medical Research, Chung Shan Medical University Hospital, Taichung, Taiwan; [4]School of Medicine, Chung Shan Medical University, Taichung, Taiwan, [5]Department of Otolaryngology, Chung Shan Medical University Hospital, Taichung, Taiwan ROC, [6]Department of Pharmaceutical & Biomedical Sciences College of Pharmacy, University of Georgia, Athens, Georgia, USA.

*To whom correspondence should be addressed:

Department of Biomedical Sciences, Chung Shan Medical University, No.110, Sec.1, Jian-guo N. Rd., Taichung, Taiwan 40201.

Fax: +886-4-23248187 Tel: +886-4-24730022, extension 11807

Email: cli@csmu.edu.tw

Supplementary Information

Supplementary Table S1. Expression values of MYCN and PRMT1 in seven neuroblastoma cell lines from experiment E-MTAB-2706

	CHP-212	KELLY	MHH-NB-11	SK-N-AS	SK-N-DZ	SK-N-FI	SK-N-SH
MYCN	2569 (657)#	2796 (710)	1009 (252)	0.8 (NA)	1566 (388)	154 (37)	4 (0.9)
PRMT1	251 (64)	366 (93)	286 (71)	255 (67)	183 (45)	197 (47)	240 (63)

#The values shown are TPM (or FPKM in parenthesis) from RNA-seq data of 675 commonly used human cancer cell lines in Expression Atlas database (https://www.ebi.ac.uk/gxa/experiments/E-MTAB-2706/Results) with gene query using MYCN and PRMT1.

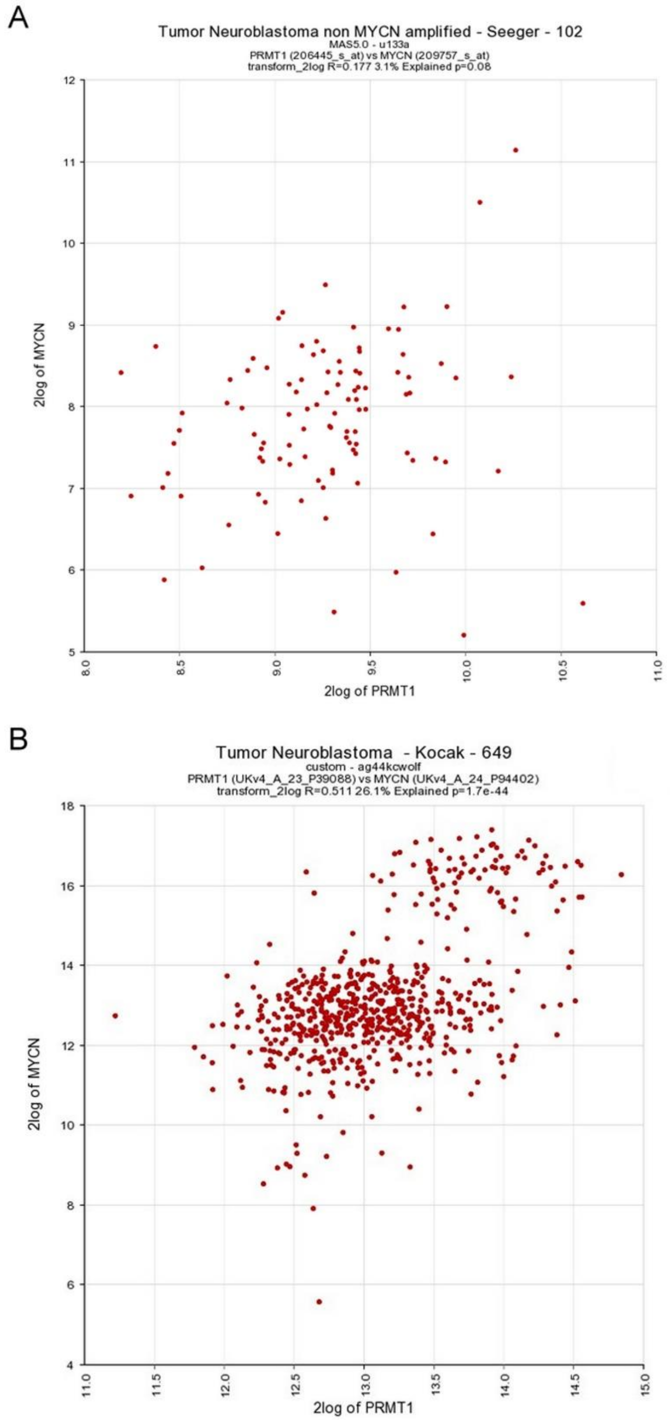

Supplementary Fig. 1. Correlation of PRMT1 and MYCN analyzed by r2 with differe nt neuroblastoma datasets. (A) XY-plot showing the correlation of PRMT1 (x-axis) and MYCN (y-axis) in non MYCN amplified Seeger dataset with 102 patients. (B) The correlation of PRMT1 (x-axis) and MYCN (y-axis) in the Kocak dataset with 476 neuroblastoma patients. The graphs were downloaded from R2 genomics analysis and visualization platform (http://r2.amc.nl).

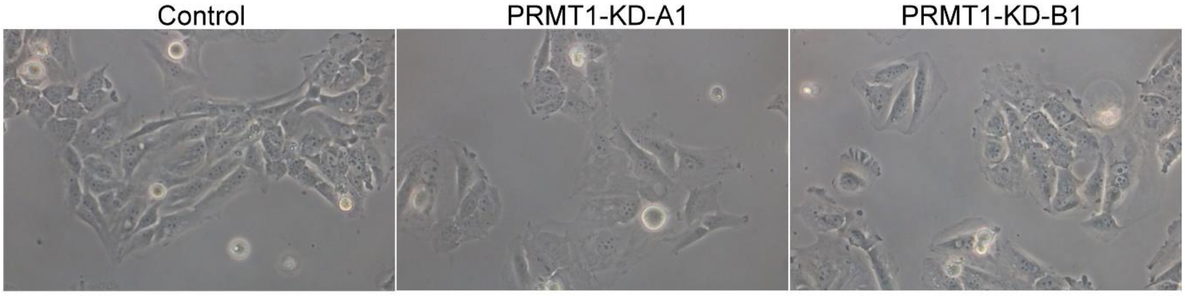

Supplementary Fig. 2.Cell morphology of control vector-infected, PRMT1 A1 or B1 shRNA-infected SK-N-AS cells.

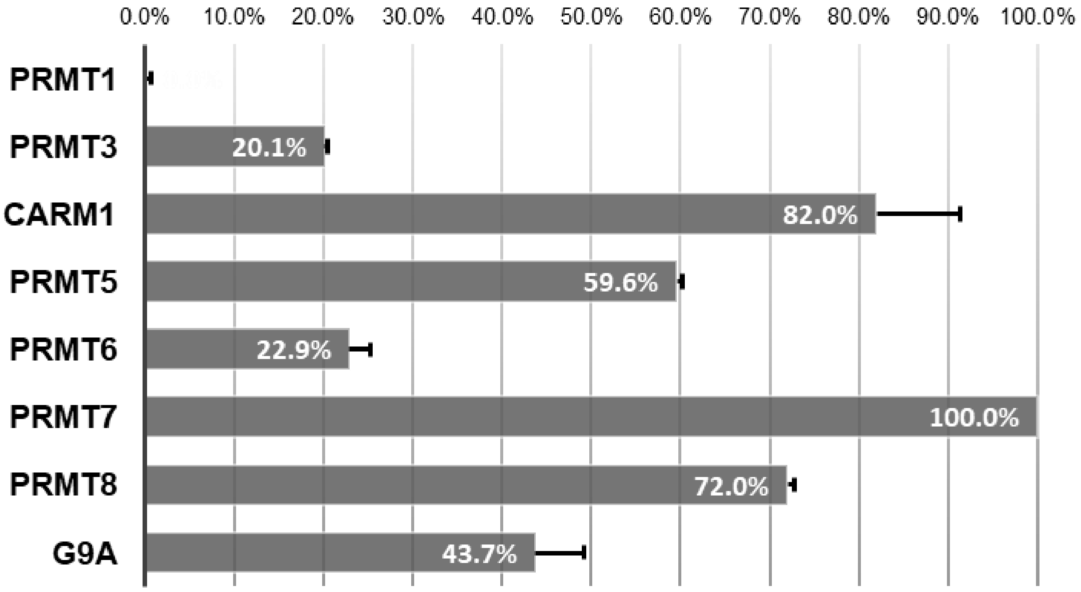

Supplementary Fig. 3. K313 selectivity panel screening. The reactions were carried out using a radiometric scintillation proximity assay (SPA). The K313 inhibitor (5 µM), biotinylated substrate peptide (1 µM), and cofactor ^{3}H-SAM (0.5 µM) were well-mixed in a reaction buffer containing 50 mM HEPES (pH 8.0), 10 mM NaCl, 0.5 mM EDTA, and 0.5 mM DTT before adding 20 nM or 40 nM of the enzyme to initiate the reaction. The reaction mixture was incubated at room temperature for 8 to 120 minutes and quenched with isopropanol, then streptavidin-coated SPA beads was added for detection. The positive control was carried out with the corresponding DMSO dilute surrogate, and the background control only contained ^{3}H-SAM, H4-20-biotin peptide and DMSO. The reported data was based on the average of two experiments.

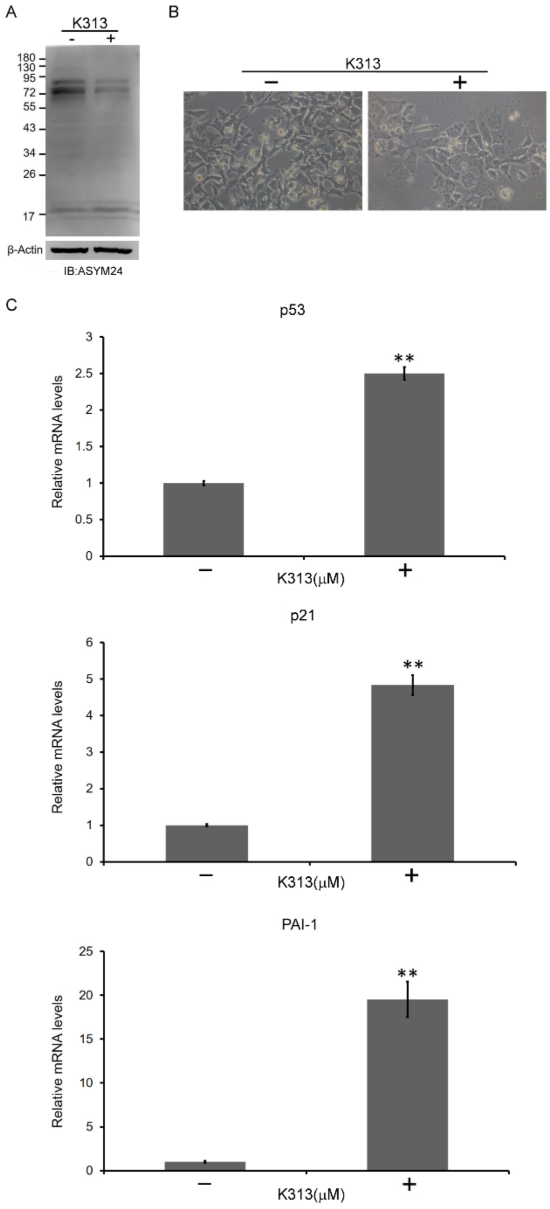

Supplementary Fig. 4. Senescent phenotypes and increased expression of *p53, p21* and *PAI-1* in SK-N-MC cells after K313 treatment.The SK-M-MC cells were treated with 2 μM of K313 for 6 days. (A) Cell extracts (20 μg of protein) from treated or untreated cells were immunoblotted with an asymmetric di-methylarginine (aDMA)-specific antibody ASYM24. Detection by anti-β-actin was used as a loading control. (B) Cell morphology of SK-N-MC cellstreated with K313 or not. (C) RNA levels of *p53, p21* and *PAI-1* in K313 treated or untreated cells determined by qRT-PCR. The data are shown as the mean±SD of triplicates. ** indicates $p<0.01$.

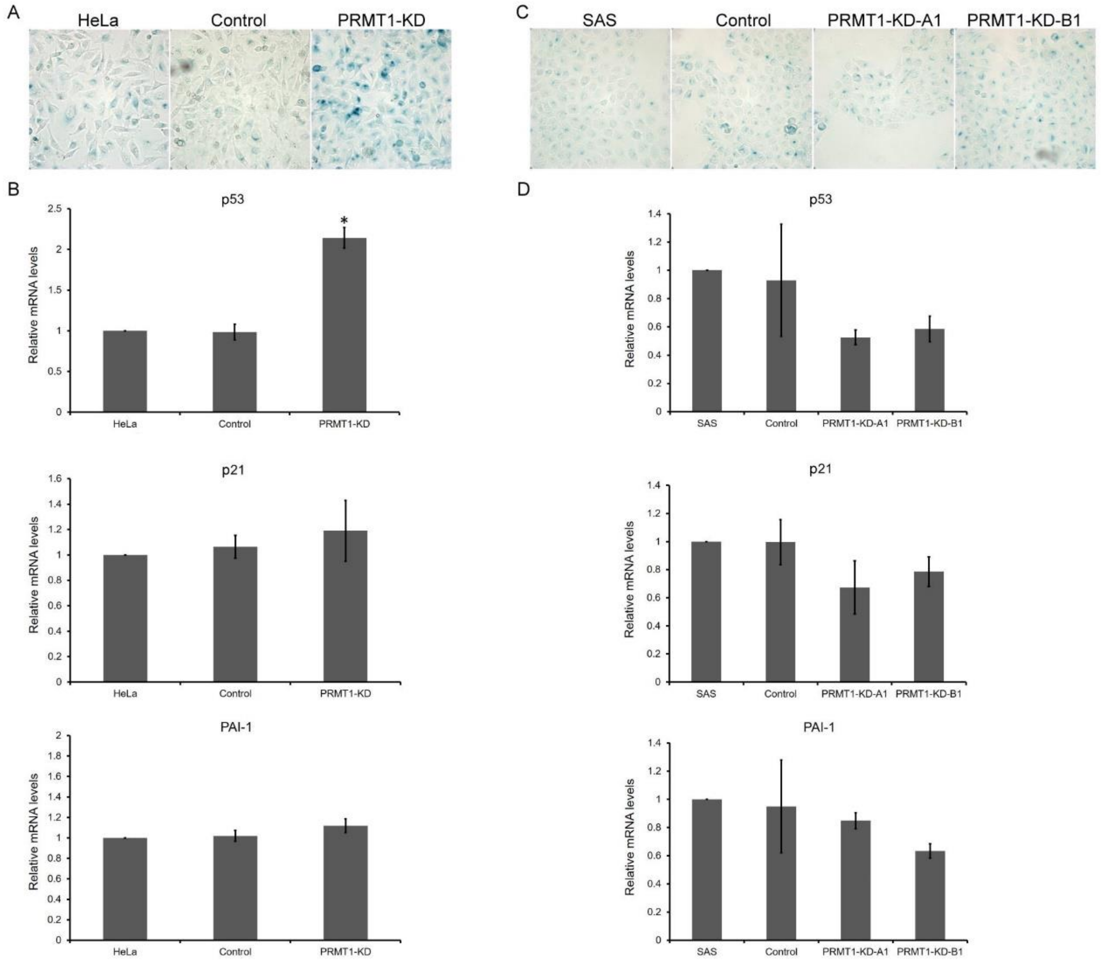

Supplementary Fig. 5. Senescent phenotypes and expression levels of p53, p21, and PAI-1 in HeLa and SAS Cells. (A) Non-infected, control vector-infected, *PRMT1* A1 plus B1 shRNA-infected HeLa cells were fixed and stained for *SA-β-Gal*. (B) RNA levels of p53, p21 and PAI-1 in PRMT1-KD HeLa cells determined by qRT-PCR. The data are shown as the mean±SD of triplicates. * indicates $p<0.05$. (C) Non-infected, control vector-infected, *PRMT1* A1 or B1 shRNA-infected SAS cells were fixed and stained for *SA-β-Gal*. (D) RNA levels of p53, p21 and PAI-1 in PRMT1-KD SAS cells determined by qRT-PCR. The data are shown as the mean±SD of triplicates.

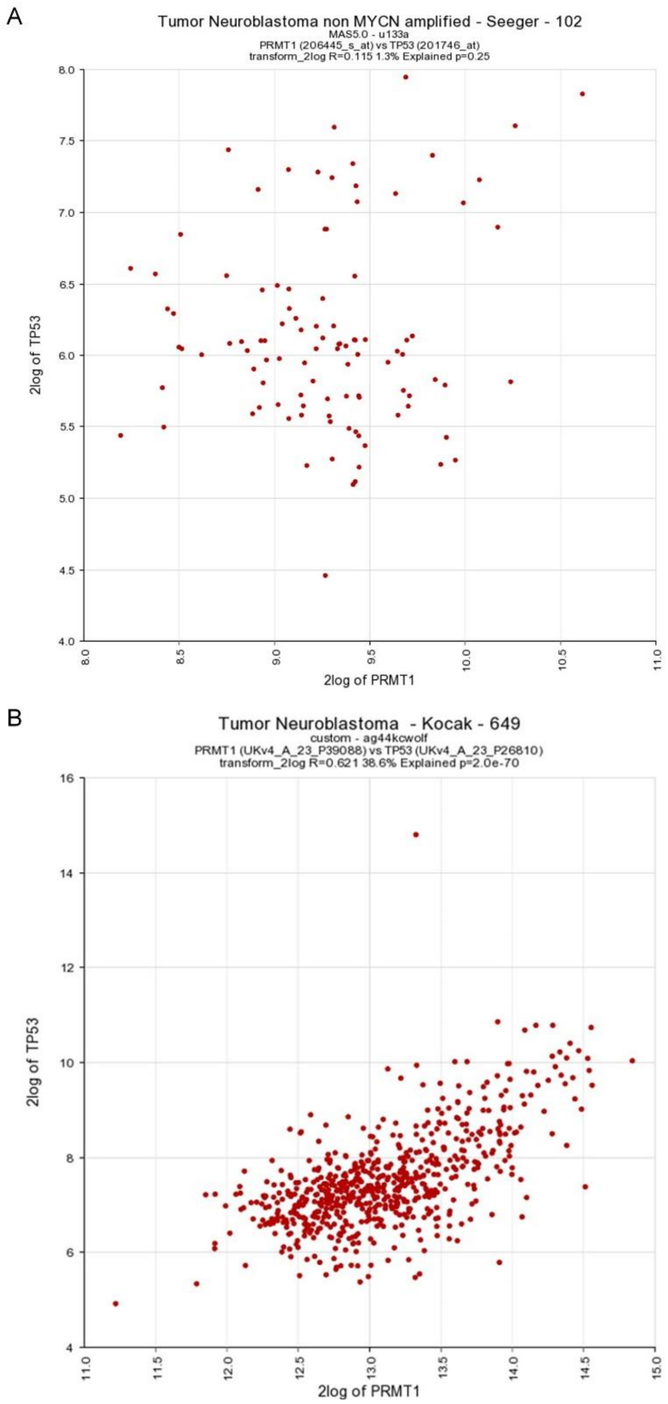

Supplementary Fig. 6. **Correlation of PRMT1 and TP53 analyzed by r2 with different neuroblastoma datasets.** (A) XY-plot showing the correlation of PRMT1 (x-axis) and TP53 (y-axis) in non MYCN amplified Seeger dataset with 102 patients. (B) The correlation of PRMT1 (x-axis) and TP53 (y-axis) in the Kocak dataset with 476 neuroblastoma patients. The graphs were downloaded from R2 genomics analysis and visualization platform (http://r2.amc.nl).

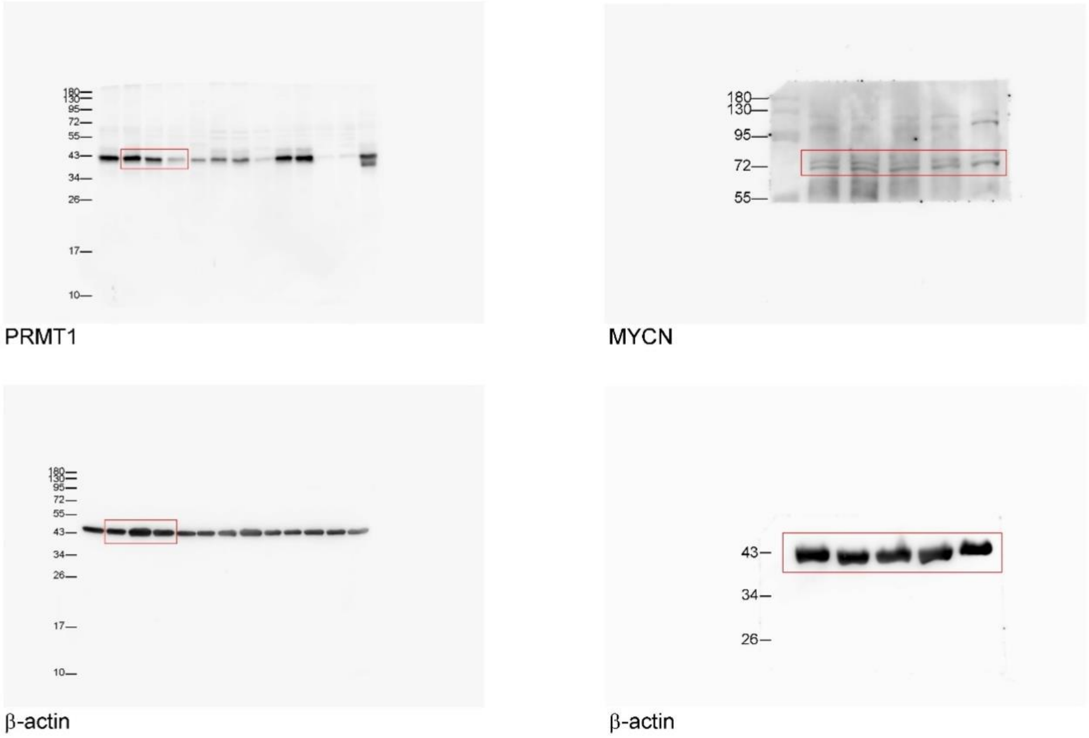

Supplementary Fig. 7. Full-size blots to Figure 1B and D. Red boxes indicate the cropped blots presented in Figure 1B and D.

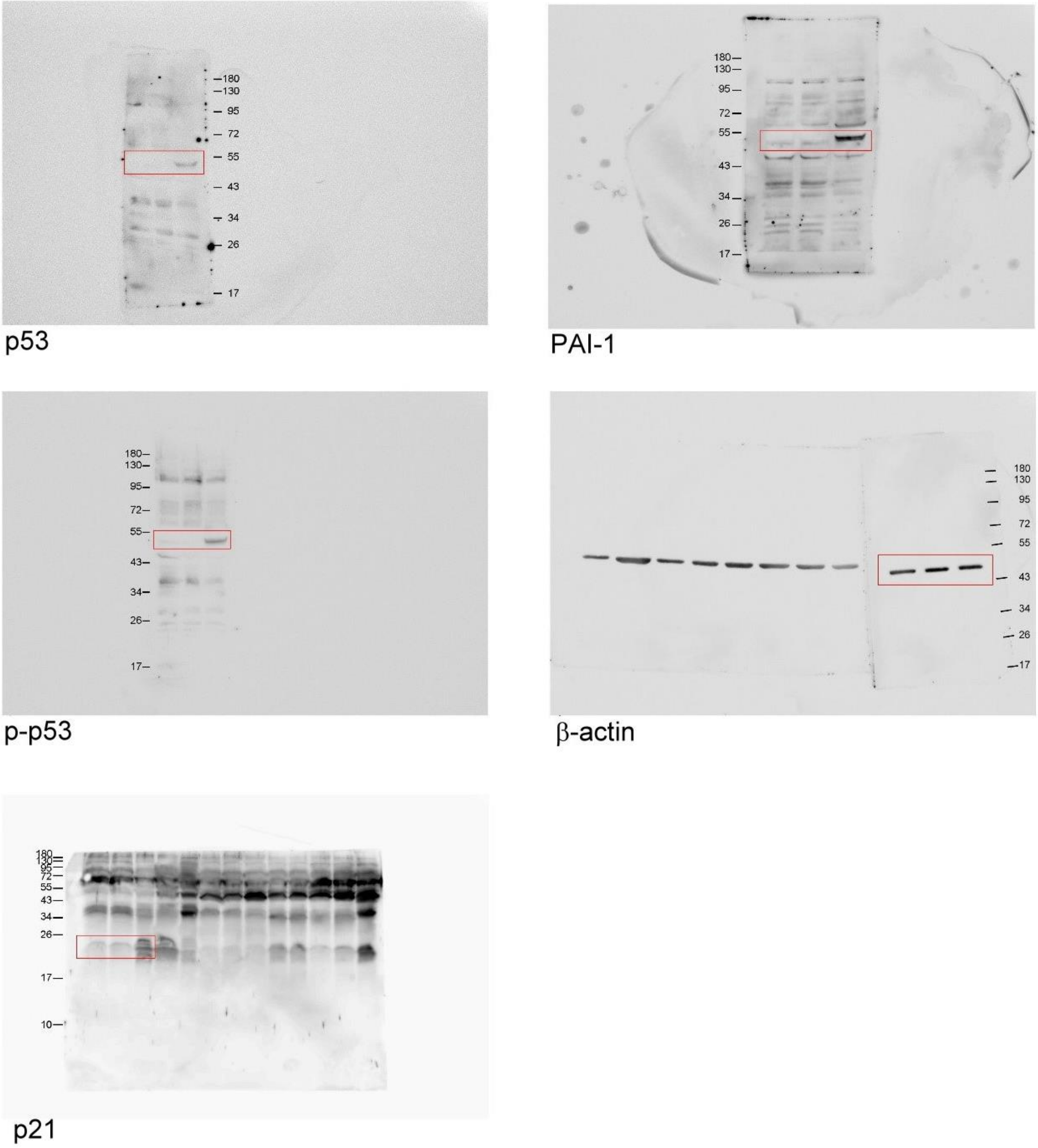

Supplementary Fig. 8. Full-size blots to Figure 3B. Red boxes indicate the cropped blots presented in Figure 3B.

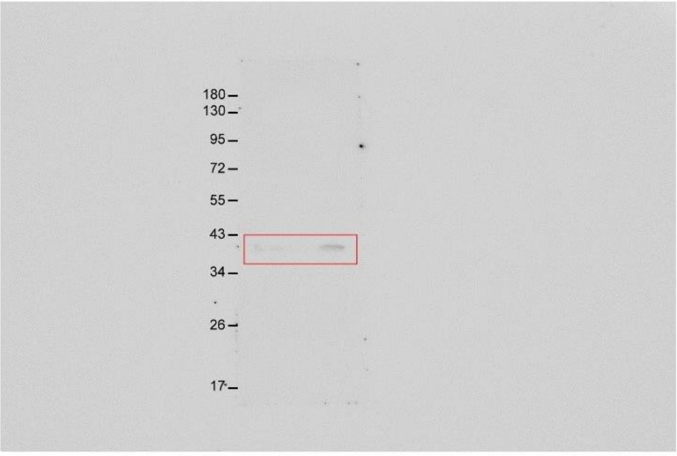

p38α

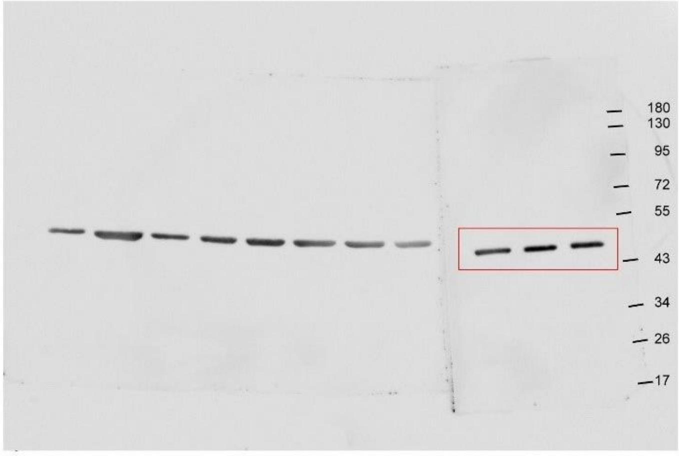

β-actin

Supplementary Fig. 9. Full-size blots to Figure 5C. Red boxes indicate thecropped blots presented in Figure 5C.

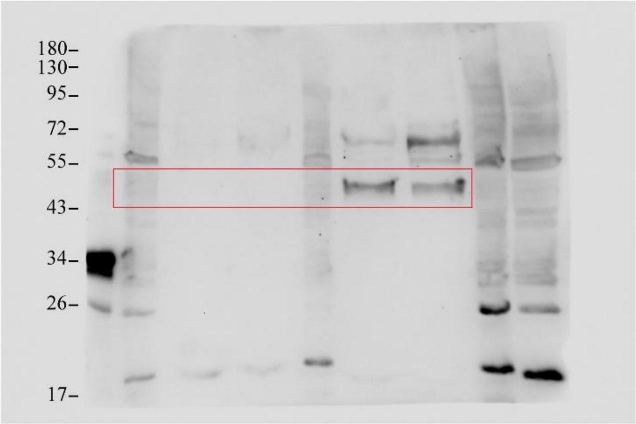

PAI-1

Supplementary Fig. 10. Full-size blots to Figure 6D. Red boxes indicate thecropped blots presented in Figure 6D.

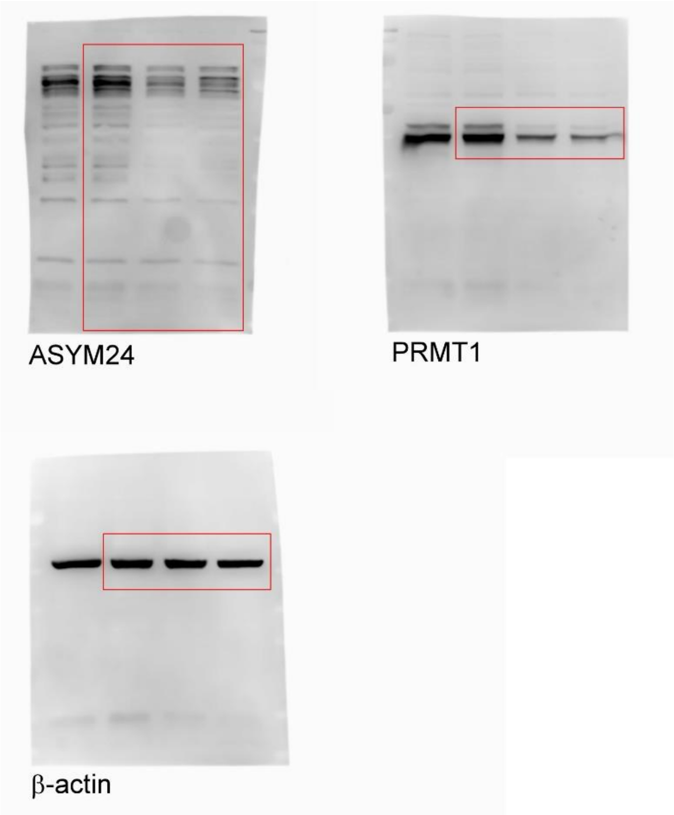

Supplementary Fig. 11. Full-size blots to Figure 7B. Red boxes indicate thecropped blots presented in Figure 7B.
